# Anti-TNF-Related Leukocytoclastic Vasculitis in Ulcerative Colitis: A Case Report

**DOI:** 10.3390/ijerph18136711

**Published:** 2021-06-22

**Authors:** Valentina Giorgio, Elisa Blasi, Donato Rigante, Cristina Guerriero, Clara De Simone, Anna Laura Fedele, Giuseppe Stella, Antonio Gasbarrini, Franco Scaldaferri

**Affiliations:** 1 Department of Life Sciences and Global Health, Fondazione Policlinico Universitario A. Gemelli IRCCS, 00168 Rome, Italy; elisa.blasi10@gmail.com (E.B.); donato.rigante@policlinicogemelli.it (D.R.); peppe.stella3@gmail.com (G.S.); 2 Università Cattolica Sacro Cuore, Fondazione Policlinico Universitario A. Gemelli IRCCS, 00168 Rome, Italy; clara.desimone@policlinicogemelli.it (C.D.S.); antonio.gasbarrini@policlinicogemelli.it (A.G.); 3 Institute of Dermatology, Fondazione Policlinico Universitario A. Gemelli IRCCS, 00168 Rome, Italy; cristina.guerriero@policlinicogemelli.it; 4 Institute of Rheumatology, Fondazione Policlinico Universitario A. Gemelli IRCCS, 00168 Rome, Italy; annalaura.fedele@policlinicogemelli.it; 5 Department of Internal Medicine, Università Cattolica Sacro Cuore, Fondazione Policlinico Universitario A. Gemelli IRCCS, 00168 Rome, Italy; franco.scaldaferri@policlinicogemelli.it

**Keywords:** leukocytoclastic vasculitis, ulcerative colitis, purpura, tumor necrosis factor

## Abstract

Background: The development of drugs directed against tumor necrosis factor (TNF)-α has dramatically modified the therapeutic approach to inflammatory bowel diseases: a larger use of such drugs has also led to a major knowledge about their adverse effects, especially on skin. The aim of this report was to describe a rare steroid-dependent form of leukocytoclastic vasculitis induced by an anti-TNF-α agent in a young woman with ulcerative colitis. Case presentation: A young girl with ulcerative colitis developed a form of leukocytoclastic vasculitis induced by an anti-TNF-α agent. Recurrent palpable purpuric lesions on her legs were the main cutaneous manifestation. Skin lesions were steroid-dependent, but improved after withdrawal of the anti-TNF-α agent and second-line immunosuppressant therapy. Conclusions: The need to develop specific recommendations to guide the use of medications for managing skin reactions induced by anti-TNF-α drugs is herein emphasized.

## 1. Introduction

The development of drugs directed against tumor necrosis factor (TNF)-α has dramatically modified the therapeutic approach to inflammatory bowel disorders, such as ulcerative colitis: first infliximab and then adalimumab were introduced in the guidelines for the treatment of refractory or difficult-to-treat inflammatory bowel diseases characterized by steroid-dependence and poor response to conventional therapies [1]. Historically, the success reported by adults on TNF inhibitors has also given children with juvenile idiopathic arthritis, uveitis, Behçet’s disease and autoinflammatory diseases the opportunity of antagonizing the effects of TNF at a clinical level [2,3,4,5,6,7,8]. Anti TNF- α, particularly adalimumab, is also used in children affected by skin diseases, like plaque psoriasis, pustular psoriasis, and hidradenitis suppurativa [9,10].

More recently, a wider use of anti-TNF-α drugs has led to major knowledge about their adverse effects, such as immune reactions that can be associated with infections, skin cancer and lymphoma [11,12].

The aim of this report was to describe a rare steroid-dependent form of leukocytoclastic vasculitis induced by an anti-TNF-α agent in a young patient with ulcerative colitis.

## 2. Case Presentation

**Figure 1:**
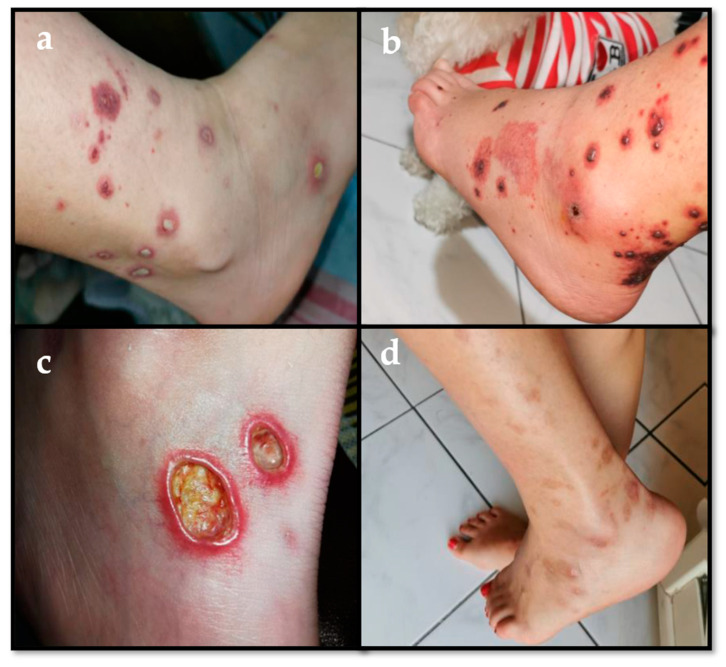
(**a**) Recurrent palpable purpuric lesions on the legs characterized by reddish-purple spots, some covered with a fibrinous film. (**b**) Swelling and palpable purpuric lesions on patient’s legs, with some haemorrhagic bullous spots. (**c**) Necrotic-ulcerative lesions on the legs, which were diagnosed as leukocytoclastic vasculitis at the skin biopsy. (**d**) Scars due to the purpuric and necrotic-ulcerative lesions on the patient’s legs.

A 13-year-old girl received the diagnosis of ulcerative pancolitis in another hospital in 2015. The first treatment used was oral mesalazine, but intermittent courses of corticosteroids were also required due to disease persistence with mesalazine only. Thereafter, an association therapy with azathioprine (AZA) was started. When the patient came to our attention in 2016, she was 14 and was in a phase of both clinical and histological remission. One year later, the patient started to complain about recurrent intestinal flares, reaching a PUCAI (Pediatric Ulcerative Colitis Activity Index) score >65, which needed intermittent prednisone/budesonide therapy, although a recurrence of symptoms was noted after their suspension. Our patient did not have any other comorbidity and no autoimmune disorder could be detected in her past history. Laboratory testing showed abnormal c-anti-neutrophil cytoplasmic antibodies (41 AU/mL) in a single determination and mild positivity of anti-nuclear antibodies (1:160, fine speckled pattern). She underwent a colonoscopy in March 2017 that showed an active intestinal disease, extended from the hepatic flexure to a proximal descending colon (Mayo 3 phase). Due to poor disease control in June 2017 the patient was started on infliximab (5 mg/kg of body weight) in addition to oral mesalazine and AZA. Four months later she presented megaloblastic anemia, a potential adverse effect with AZA, despite ongoing acid folic therapy at 5 mg/day. AZA was promptly discontinued with rapid resolution of anemia. Since no remission was reached and a PUCAI score of 30–50 persisted, the patient needed to modify infliximab (IFX) therapy in terms of dosage and frequency of infusions. Initially she received IFX every 2–4–8 weeks at the standard dose of 5 mg/kg. Due to absent clinical response, inadequate IFX blood levels and absence of anti-IFX antibodies, the schedule was changed to 10 mg/kg every 8 weeks. After 4 months, the drug was undetectable and anti-drug antibodies were absent, so the frequency of IFX administrations was shortened to 4 weeks, reaching an adequate level of blood IFX without any anti-drug antibodies. After one year on IFX, the girl developed psoriasis-like lesions on the scalp and trunk combined with a papulo-pustular rash on the abdomen and legs. The latter were biopsied, revealing a likely reaction to IFX. This cutaneous picture was treated with topical corticosteroids and recovered. Later on, the patient presented recurrent palpable purpuric lesions on her legs characterized by reddish-purple spots, some covered with a fibrinous film (Figure 1a). Laboratory tests were performed, showing no anemia and normal white blood cell count; inflammatory markers were normal as well as liver and renal function. The whole autoimmunity panel (p-anti-neutrophil cytoplasmic antibodies, c-anti-neutrophil cytoplasmic antibodies, antinuclear antibodies, anti-extractable nuclear antigen antibodies panel (anti-Sjögren syndrome A and B, Smith antibody and anti-ribonucleoprotein), anti-dsDNA, rheumatoid factor, cryoglobulins, C3c, C4, lupus anticoagulant, anti-cardiolipin and anti-beta 2-glycoprotein-I antibodies) was unrevealing. Screening for infections was unremarkable. Urinalysis was negative for proteinuria and hematuria. The only abnormal finding, but without any pathological meaning, was a mild elevation of total serum IgA (4.9 g/L, n.v. 0.6–3.48). Chest X-ray film, renal ultrasound assessment, whole body-magnetic resonance imaging excluded any extra-intestinal disease and/or joint involvement.

An alternative biological therapy was needed: vedolizumab was considered, but was disregarded as off-label in pediatrics. Therefore, in October 2019, a switch to adalimumab was established, starting with a dose of 160 mg subcutaneously (sc) followed by 80 mg sc after 2 weeks (induction) and then continued with 40 mg sc every 2 weeks (maintenance), which led to a definite intestinal remission. Unfortunately, skin lesions seemed to persist: in fact, the patient had relapsing phases of psoriasis on the scalp and relapsing palpable purpuric with hemorrhagic bullous lesions on the legs (Figure 1b) combined with dysestesia. A biopsy of these latter lesions revealed leukocytoclastic vasculitis. Electromyography was negative for neuropathy. A colonoscopy was performed and showed an endoscopic intestinal remission.

Accordingly, in consideration of the persistent skin lesions, adalimumab was stopped and the anti-interleukin-12/23 monoclonal antibody ustekinumab (recently introduced in dermatology, especially for psoriasis) started at a dosage of 90 mg every 8 weeks. However, no beneficial effects were seen. In fact, after 15 days from ustekinumab introduction, the purpuric rash turned to necrotic-ulcerative lesions (Figure 1c), and a new punch biopsy was performed, confirming the diagnosis of leukocytoclastic vasculitis.

**Figure 2:**
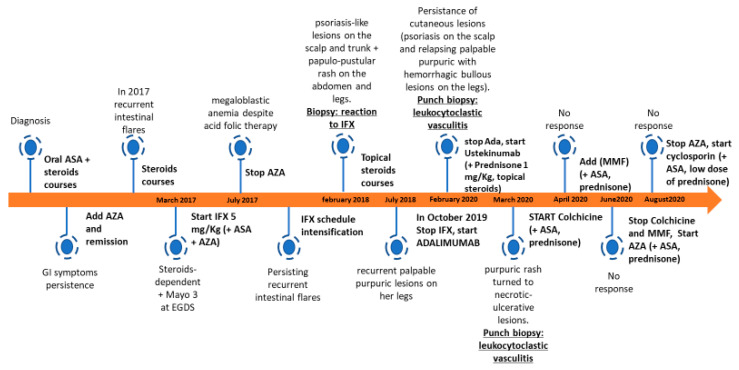
Timeline of patient’s diagnostic process and treatment. ASA, aminosalicylate; AZA, azathioprine; IFX, infliximab; MMF, mycophenolate mofetil.

Prednisone at the dose of 1 mg/kg/day combined with colchicine at the dose of 0.5 mg twice a day were started. After the first three months, colchicine efficacy was lost, and mycophenolate mofetil (500 mg twice a day) and AZA (75 mg/day) were started. Due to poor efficacy, mycophenolate mofetil and AZA were stopped after two weeks. As purpura and ulcerative lesions persisted, the whole autoimmunity panel was repeated but was unremarkable. Therefore, cyclosporin at the dose of 200 mg/day was introduced, finally leading to an overall improvement of the skin picture. The patient is currently well, with non-active scars at her lower limbs (Figure 1d), and keeps on receiving cyclosporine plus low-dose prednisone (17.5 mg/day). A timeline of the patient’s diagnostic process and treatment is shown in Figure 2.

## 3. Discussion and Conclusions

Leukocytoclastic vasculitis is a hypersensitivity-induced inflammation of vessels in which drugs can be recognized to be causative in approximately 10–24% of cases [13]. Few studies have reported vasculitides induced by anti-TNF-α agents in different populations of patients, mostly including rheumatoid arthritis for adults, but also juvenile idiopathic arthritis in children. In a review by Ramos-Casals et al., 113 cases of vasculitides were reported during therapy with TNF-α inhibitors. The underlying condition was rheumatoid arthritis in 84%, Crohn’s disease in 6%, juvenile idiopathic arthritis in 4%, and other disorders in 6%. Etanercept was involved in 52% of such cases, IFX in 42% and adalimumab in 4%. The characteristics of cutaneous lesions were purpura in 57%, ulcerative lesions in 9%, nodules in 9%, digital vasculitis in 6% and maculo-papular rash in 5%. Moreover, extra-cutaneous manifestations were observed in 24% of patients, mostly peripheral neuropathy and renal vasculitis. The majority of patients (89%) responded to withdrawal of the specific TNF-α inhibitor, while corticosteroids were needed in 25% of them and immunosuppressant agents in 15%. Interestingly, 11% of cases did not require the discontinuation of the biological treatment [14].

In another study conducted by Saint Marcoux et al. describing 39 cases of vasculitides induced by anti-TNF-α agents, six patients (15%) were able to continue biologic therapy and the resolution of vasculitis was complete [15].

In a retrospective review by Sokumbi et al., eight patients with anti-TNF-induced vasculitis had a mean time of 6.9 months up to the resolution of vasculitis after discontinuing drugs, but all received prednisone and seven also received immunosuppressant agents [16].

Only a few cases of anti-TNF-induced vasculitis have been related to inflammatory bowel disease [17,18,19,20,21,22]. Unfortunately, no guidelines are available for the management of this condition.

The pathogenesis of anti-TNF-related leukocytoclastic vasculitis is also poorly known, though immune complexes including the anti-TNF antibody which precipitate on the walls of little vessels are presumed to induce a local activation of the complement system and a type III hypersensitivity reaction. Other explanations might be a cytokine imbalance due to TNF-α suppression, with predominance of the Th2 pattern, but also drug toxicity, exerting direct vascular damage [14,23,24].

Most patients with only skin involvement can be treated in an outpatient setting, with resolution of symptoms obtained within days to months. Some authors described the possibility to treat this condition by simply withdrawing the anti-TNF-α agent or reducing its dosage. Conversely, the most complicated pictures displaying systemic manifestations and/or extra-intestinal involvement might require a more aggressive treatment.

A recent case reported by Bouhuys et al. described a 16-year-old girl with active ulcerative colitis, who presented anti-neutrophil cytoplasmic antibody-associated leukocytoclastic vasculitis with involvement of peripheral nerves while on therapy with mesalazine and AZA, worsened after the introduction of IFX infusions [25]. While the role of inflammatory bowel disease itself in the development of skin diseases (such as erythema nodosum, pyoderma gangrenosum and psoriasis) is well known [26,27], few cases of leukocytoclastic vasculitis have been reported for young patients with inflammatory bowel disease, occurring during both active colitis and quiescence, but without a relationship with the extension of intestinal involvement [28]: therapy with corticosteroids and immunosuppressant agents usually leads to the resolution of skin lesions in such patients [25,29].

Our patient started to present skin lesions referred to a leukocytoclastic vasculitis 1 year after starting IFX infusions, but also during an active phase disease: lesions persisted after reaching clinical and histological intestinal remission with adalimumab. Only the discontinuation of the biological therapy in association with corticosteroids and immunosuppressants led to the final recovery of cutaneous lesions, supporting the hypothesis of a drug-related skin disease.

Hence, in conclusion, considering the lack of medical literature upon this field, more studies and discussion are needed in order to develop specific recommendations guiding the use of corticosteroids and immunosuppressant agents in the management of vasculitides likely induced by anti-TNF drugs.

## Data Availability

The data that support the findings of this study are available from the corresponding author, upon reasonable request.
